# Catheter-related Bacteremia and Multidrug-resistant *Acinetobacter lwoffii*

**DOI:** 10.3201/eid1302.060858

**Published:** 2007-02

**Authors:** Luciano Tega, Katia Raieta, Donatella Ottaviani, Gian Luigi Russo, Giovanni Blanco, Antonio Carraturo

**Affiliations:** * Ospedale Santa Maria Goretti, Latina, Italy; † Istituto di Scienze dell’Alimentazione, Avellino, Italy; ‡ Istituto Zooprofilattico Sperimentale dell’Umbria e delle Marche, Ancona, Italy

**Keywords:** Acinetobacter lwoffii, catheter-related bacteremia, immunocompromised patients, antimicrobial drug resistance, PFGE, antibiotyping, cross-transmission, multidrug resistance, letter

**To the Editor:** *Acinetobacter* species are ubiquitous in the environment. In recent years, some species, particularly *A*. *baumannii*, have emerged as important nosocomial pathogens because of their persistence in the hospital environment and broad antimicrobial drug resistance patterns (*1*,*2*). They are often associated with clinical illness including bacteremia, pneumonia, meningitis, peritonitis, endocarditis, and infections of the urinary tract and skin (*3*). These conditions are more frequently found in immunocompromised patients, in those admitted to intensive care units, or in those who have intravenous catheters, and those who are receiving mechanical ventilation (*4*,*5*).

The role of *A*. *baumannii* in nosocomial infections has been documented (*2*), but the clinical effect of other *Acinetobacter* species has not been investigated. *A*. *lwoffii* (formerly *A*. *calcoaceticus* var. *lwoffii*) is a commensal organism of human skin, oropharynx, and perineum that shows tropism for urinary tract mucosa (*6*). Few cases of *A*. *lwoffii* bacteremia have been reported (*3*,*5*–*7*). We report a 4-year (2002–2005) retrospective study of 10 patients with *A*. *lwoffii* bacteremia admitted to a 600-bed teaching hospital in central Italy.

All 10 patients were immunocompromised; 8 had used an intravascular catheter (peripheral or central) and 2 had used a urinary catheter. Blood cultures of the patients were analyzed with the BacT/ALERT 3D system (bioMérieux, Marcy l’Etoile, France). Isolates were identified as *A*. *lwoffii* by using the Vitek 2 system and the API 20NE system (both from bioMérieux).

Susceptibilities of 10 *A*. *lwoffii* isolates to 18 antimicrobial drugs were determined by the broth microdilution method, according to Clinical and Laboratory Standards Institute (CLSI, formerly NCCLS) guidelines (*8*). The drugs tested were amikacin, ampicillin-sulbactam, aztreonam, cefepime, cefotaxime, ceftazidime, ceftriaxone, ciprofloxacin, gentamicin, imipenem, levofloxacin, meropenem, ofloxacin, piperacillin, piperacillin-tazobactam, tetracycline, tobramycin, and trimethoprim-sulfamethoxazole. MIC was defined as the lowest drug concentration that prevented visible bacterial growth. Interpretative criteria for each drug tested were as in CLSI guidelines (*8*). *A*. *lwoffii* resistant to >4 classes of drugs were defined as multidrug-resistant (MDR) isolates.

*A*. *lwoffii* isolates were genotyped by pulsed-field gel electrophoresis (PFGE) to determine their epidemiologic relatedness. Chromosomal DNA was digested with *Sma*I (*9*) and analyzed with a CHEF DR II apparatus (Bio-Rad Laboratories, Hercules, CA, USA). PFGE patterns were classified as identical, similar (differed by 1–3 bands), or distinct (differed by >4 bands) (*10*).

Among the 10 *A*. *lwoffii* isolates, 6 were susceptible to all drugs except cephalosporins (cefepime, cefotaxime, ceftazidime, and ceftriaxone) and aztreonam. The other 4 isolates were MDR: 3 were susceptible only to imipenem (MICs 1–4 μg/mL), meropenem (MICs 1–2 μg/mL), and amikacin (MICs 2–4 μg/mL). The fourth MDR strain was susceptible to imipenem (MIC 2 μg/mL), meropenem (MIC 2 μg/mL), amikacin (MIC 4 μg/mL), and ciprofloxacin (MIC 1 μg/mL). Seven antimicrobial drug resistance profiles were detected (Table).

**Table:** Antimicrobial drug susceptibility and pulsed-field gel electrophoresis (PFGE) patterns of 10 *Acinetobacter lwoffii* strains, Italy, 2002–2005

No.	Source*	Drug†																		Antibiotype	PFGE
		AS	PI	PT	CE	CT	CA	CF	AT	CI	LE	OF	GM	TM	AM	TC	IP	MP	TS		
1	ICU	R	R	R	R	R	R	R	R	R	R	R	R	R	S	R	S	S	R	a	A
2	ICU	R	R	R	R	R	R	R	R	R	R	R	R	R	S	R	S	S	R	a	B
3	OW	R	R	R	R	R	R	R	R	R	R	R	R	R	S	R	S	S	R	a	B
4	ICU	R	R	R	R	R	R	R	R	S	R	R	R	R	S	R	S	S	R	b	C
5	ICU	S	S	R	S	S	R	R	R	R	R	R	S	S	S	S	S	S	S	c	D
6	ICU	R	S	S	S	R	R	R	S	S	S	S	R	R	S	S	S	S	S	d	E
7	UW	S	S	S	R	R	R	R	R	S	S	S	S	S	S	R	S	S	S	e	F
8	ICU	S	R	S	R	R	S	R	R	S	S	S	S	S	S	S	S	S	R	f	G
9	ICU	S	R	S	R	R	S	R	R	S	S	S	S	S	S	S	S	S	R	f	G
10	MW	S	S	S	S	S	R	S	R	S	R	R	R	S	R	S	S	S	S	g	H

*ICU, intensive care unit; OW, orthopedic ward; UW, urologic ward; MW, medical ward. †AS, ampicillin-sulbactam; PI, piperacillin; PT, piperacillin-tazobactam; CE, cefepime; CT, cefotaxime; CA, ceftazidime; CF, ceftriaxone; AT, aztreonam; CI, ciprofloxacin; LE, levofloxacin; OF, ofloxacin; GM, gentamicin; TM, tobramycin; AM, amikacin; TC, tetracycline; IP, imipenem; MP, meropenem; TS, trimethoprim-sulfamethoxazole; R, resistant; S, susceptible.

Macrorestriction analysis of the *A*. *lwoffii* isolates identified 8 distinct PFGE types. Two MDR strains (strains 2 and 3 in the Table), which were isolated from patients in different wards, and 2 non-MDR strains (strains 8 and 9), which were isolated from patients in the same ward, had similar PFGE patterns and identical resistance phenotypes. These findings suggest nosocomial transmission. Nine of the 10 patients survived after catheter removal or treatment with appropriate antimicrobial drugs. These results confirm that catheter-related *A*. *lwoffii* bacteremia in immunocompromised hosts is associated with a low risk for death (*4*,*6*).

This study identified *A*. *lwoffii* MDR strains that cause bacteremia in immunocompromised catheterized patients. Our data are consistent with those of previous reports on the role of catheters as the principal source of *A*. *lwoffii* infections.
